# Spectral-Based Classification of Genetically Differentiated Groups in Spring Wheat Grown under Contrasting Environments

**DOI:** 10.3390/plants12030440

**Published:** 2023-01-18

**Authors:** Paulina Ballesta, Carlos Maldonado, Freddy Mora-Poblete, Daniel Mieres-Castro, Alejandro del Pozo, Gustavo A. Lobos

**Affiliations:** 1 Instituto de Nutrición y Tecnología de Los Alimentos, Universidad de Chile, Santiago 7830490, Chile; 2 Centro de Genómica y Bioinformática, Facultad de Ciencias, Universidad Mayor, Santiago 8580745, Chile; 3 Institute of Biological Sciences, University of Talca, Talca 3460000, Chile; 4 Plant Breeding and Phenomic Center, Faculty of Agricultural Sciences, University of Talca, Talca 3460000, Chile

**Keywords:** remote sensing classification, genetic structure, *Triticum aestivum*, foliar reflectance

## Abstract

The global concern about the gap between food production and consumption has intensified the research on the genetics, ecophysiology, and breeding of cereal crops. In this sense, several genetic studies have been conducted to assess the effectiveness and sustainability of collections of germplasm accessions of major crops. In this study, a spectral-based classification approach for the assignment of wheat cultivars to genetically differentiated subpopulations (genetic structure) was carried out using a panel of 316 spring bread cultivars grown in two environments with different water regimes (rainfed and fully irrigated). For that, different machine-learning models were trained with foliar spectral and genetic information to assign the wheat cultivars to subpopulations. The results revealed that, in general, the hyperparameters ReLU (as the activation function), adam (as the optimizer), and a size batch of 10 give neural network models better accuracy. Genetically differentiated groups showed smaller differences in mean wavelengths under rainfed than under full irrigation, which coincided with a reduction in clustering accuracy in neural network models. The comparison of models indicated that the Convolutional Neural Network (CNN) was significantly more accurate in classifying individuals into their respective subpopulations, with 92 and 93% of correct individual assignments in water-limited and fully irrigated environments, respectively, whereas 92% (full irrigation) and 78% (rainfed) of cultivars were correctly assigned to their respective classes by the multilayer perceptron method and partial least squares discriminant analysis, respectively. Notably, CNN did not show significant differences between both environments, which indicates stability in the prediction independent of the different water regimes. It is concluded that foliar spectral variation can be used to accurately infer the belonging of a cultivar to its respective genetically differentiated group, even considering radically different environments, which is highly desirable in the context of crop genetic resources management.

## 1. Introduction

The frequency and severity of drought events have been increasing in recent years in various regions around the world [1], a trend that is expected to continue as a consequence of climate change [2]. These drought events are responsible for much of the loss of crop productivity, varying between 10% and 35%, depending on its intensity and duration [3]. Several mechanisms that could provide tolerance to drought stress in agricultural crops have been proposed. For instance, Hassanisaadi et al. [4] notes that nanoparticles have protective effects against drought stress, while Van Oosten et al. [5] indicate that biostimulators and bioeffectors improve plant resilience in water-limiting environments. However, a better understanding of the specific mechanisms of action of these molecules is necessary if we want to improve the within-field crop yield in water stress, which suggests that more studies are needed in this direction [4,5]. On the other hand, previous studies have discussed how the water deficit interferes with damage at critical phenological stages of cereal growth (i.e., grain filling, plant emergence, and pollination) and generates potential noise in phenotypic measurements [6,7], which affects the selection and classification of productive genotypes. Therefore, in order to ensure food security, several breeding programs not only focus on the identification of mechanisms against drought stress but also on the identification of models that, regardless of environmental conditions, help in the selection of genotypes that are productive through phenotypic measurements.

The genetic structure of cereal germplasms has been extensively evaluated using highly polymorphic molecular markers, such as Single Nucleotide Polymorphisms (SNPs), Diversity Arrays (DArTs), and Simple Sequence Repeats (SSR) [8,9,10]. These studies consist of grouping individuals and identifying genetically differentiated groups through the similarity between their allele frequencies. On the other hand, the individuals of a population can not only be classified by the neutral variation of the genome but also by phenotypic, ecological, and behavioral criteria, which are required to be evaluated in terms of their correspondence with the genetic patterns detected in the populations [11].

High-throughput plant phenotyping (HTPP) techniques have been proposed as a fast and low-cost alternative for measuring complex traits, such as yield and resistance to stress factors (biotic and abiotic) under field and greenhouse conditions [12,13]. In fact, HTPP platforms started to be routinely used in plant breeding programs, characterizing genotypic variability at the canopy level under unfavorable growth conditions [11,14,15]. Current methodologies include the use of different portable sensors that provide readings of spectral reflectance, leaf temperature, RGB images, fluorescence, and other techniques for obtaining the 3D volume of each plot. Theoretically, the use of non-proximal sensors allows for the phenotyping of thousands of individuals in one day, which has an impact similar to that achieved by high-throughput DNA sequencing technologies [11,12,13,14,15].

Different analytical models have been used to evaluate the classificatory capacity of spectral data, with supervised methods being the most used. For instance, Partial least squares-discriminant analysis (PLS-DA) is one of the most widely used classification algorithms to deal with the multicollinearity problem of high-dimensional spectral data [16]. This method has been used in the context of genotype classification, disease detection, and the physiological states of crops [16,17,18]. On the other hand, Artificial Neural Networks (ANNs) have emerged as a powerful tool for the predictive modeling of big data. Moreover, machine learning approaches such as Multi-Layer Perceptron (MLP) and Convolutional Neural Network (CNN) have been shown to be superior to PLS-DA in classifying omics, phenotypic, and spectral data [11,19]. The big drawback is that many of these methods (CNN and MLP), in contrast to the measure-based methods (such as FlowerMorphology morphometry software [20]), are “black boxes”, so they do not disclose all feature interactions and do not provide classification rules, and the results referred to can hardly be understood by the user [21]. However, ANNs are flexible structured mathematical tools that can create a non-linear mapping between input and output information spaces [22], allowing for the adjustment of a series of parameters to increase their classification capacity (e.g., number of layers and activation functions, among others). Furthermore, ANNs can learn, identify, and model highly complex nonlinear relationships in biological systems, which provides a predictive and classification power superior to traditional statistical approaches [23].

Due to the importance of the efficient management of genetic resources for the enhancement of agricultural crop resilience to challenges of climate change, we present, in this article, a spectral-based classification of genetically differentiated groups in spring wheat, which is useful even considering radically different environments that differ in water availability during the growing season. For this, different machine-learning models were trained with foliar spectral information to assign wheat cultivars to subpopulations previously defined using SNPs markers.

## 2. Results

This section shows the wheat spectral-based classification accuracy obtained from three machine-learning-based methods (PLS-DA, MLP, and CNN). The workflow considered three steps: (I) the estimation of the genetic population structure (Section 2.1), (II) the identification of better hyperparameter combinations in the neural network models (MLP and CNN) (Section 2.2), and (III) the comparison of neural network models with PLS-DA (Section 2.3).

### 2.1. Genetic Structure Analysis and Spectral Data

**Figure 1:**
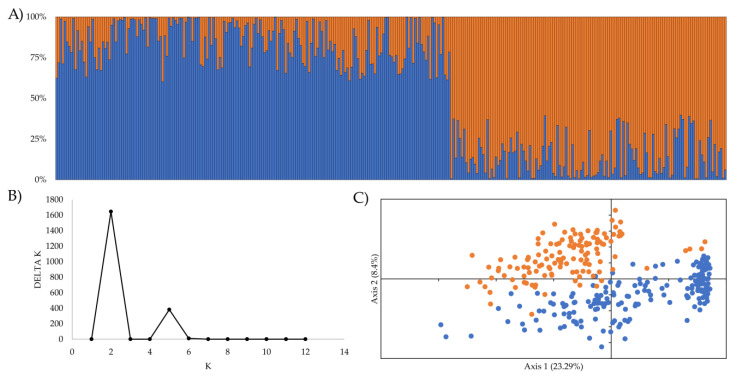
Population structure analysis for 316 spring wheat cultivars. (**A**) Genetic structure inferred by a Bayesian clustering model using STRUCTURE. The proportion of colored segments (light blue and orange) indicates the proportion of the genome extracted from the two sub-groups (cluster 1 and cluster 2, respectively). (**B**) Delta K values for different numbers of populations assumed (K) in the STRUCTURE analysis. (**C**) Principal Component Analysis (PCA) shows the 316 cultivars spatially distributed in relation to the first two main components. The cultivars were colored according to cluster matching the structure population (blue for cluster 1 and orange for cluster 2).

The genetic population structure analysis indicated that the most probable number of subpopulations in the sample was two (K = 2), according to the methodology of Evanno et al. [24] (Figure 1A,B). These results were similar to what was visualized with the first two primary components of the PCA method (Figure 1C), which, in the axes PC1 and PC2, explained 23.29% and 8.4% of the variation in the genotypic data, respectively. Moreover, Bayesian credibility intervals (at 95%) of the FST values confirm the existence of significant differences between both clusters. The estimates of the FST for clusters 1 and 2 were 0.1820 (0.1650–0.1980) and 0.3338 (0.3110–0.3530), respectively. Cluster 1 contained 59% of cultivars (186/316), of which 70%, 23% and 7% corresponded to the CIMMYT, INIA-Chile, and INIA-Uruguay breeding programs, respectively. On the other hand, cluster 2 consisted of 41% (130/316), including mainly INIA-Uruguay cultivars (96%).

**Figure 2:**
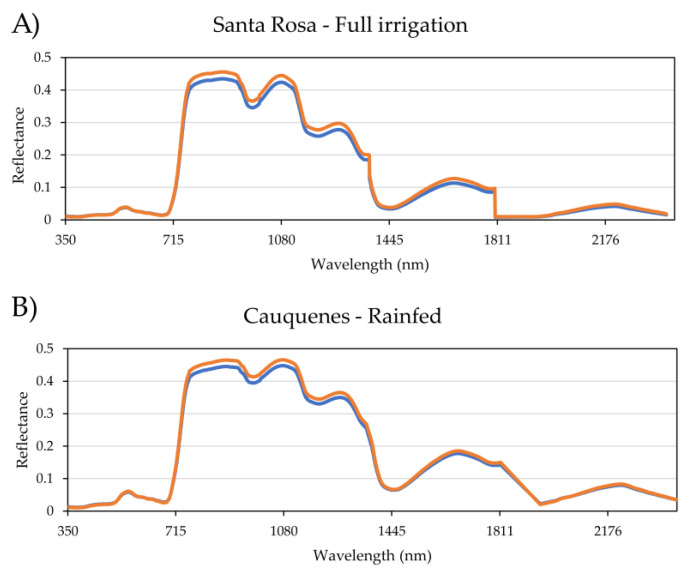
The average of spectral curves for each genetically differentiated group (Q1, in blue, and Q2, in orange) in the environments Santa Rosa (**A**) and Cauquenes (**B**).

In Figure 2, a comparison between the average of spectral curves produced by each genetically differentiated group in each environment is shown. The principal wavelength that distinguishes both groups is between 741 and 1357 nm, in which Santa Rosa presents greater differences in the wavelengths than Cauquenes (0.020–0.017 nm, respectively).

### 2.2. Hyperparameter Evaluation

For both neural network models (CNN and MLP), the hyperparameter combinations depicted in Table 1 were evaluated. The evaluation was carried out by running the search for the best classification accuracy over the defined hyperparameters; therefore, for each combination of hyperparameters, both neural networks were trained. On average, the ReLU activation function was significantly more accurate (CA = 0.80–0.84) than other activation functions in assigning the individuals to their respective clusters, considering both neural networks in both environments—Santa Rosa and Cauquenes (Table 1 and Table 2). On the other hand, the Adam optimizer had a higher CA than rmsprop in both environments; nonetheless, in the full irrigation site (Santa Rosa), the adam was significantly more efficient in correctly assigning individuals to clusters. In general, both the batch size and the number of layers did not have a significant effect on the ability of CNN and MLP to assign individuals to the respective clusters of each environment evaluated (Table 1 and Table 2).

Both artificial neural networks showed the highest CA with the following combination of hyperparameters: the ReLU activation function, the adam optimizer, a batch size of 10, and only one conv1D layer. However, the MLP network in the Cauquenes environment showed an increase in the assignment of individuals when the batch size changed from 10 (CA = 0.78) to 20 (CA = 0.88). For both the MLP and CNN neural networks, the classification accuracy was lower for the site with a low irrigation supply (Cauquenes). Nevertheless, the CNN had a higher CA value than MLP in the prediction of the genetic structure from both environments.

### 2.3. Best Performing Models

The best combination of hyperparameters seen in the previous section was used to train the CNN and MLP final model and obtain a comparison of their performance with PLS-DA. The performance of the best MLP and CNN models with their hyperparameters achieved a CA of 0.88 (MLP in Cauquenes) and 0.93 (CNN in Santa Rosa), while PLS-DA showed the lowest CA values in both environments. In general, MLP was as efficient as CNN in assigning individuals to cluster 1; however, CNN exhibited a significantly higher effectiveness than MLP in the allocation of individuals to cluster 2 for both environments. Moreover, CNN exhibited the highest CA for both environments, which was significantly high compared to that of other models (Table 3). Interestingly, PLS-DA showed significantly higher CA values in Cauquenes (rainfed) than in Santa Rosa (full irrigation), while both neural network models exhibited higher CA values in Santa Rosa; however, CNN was not statistically different between both environments. This indicates that CNN and PLS-DA models are suitable for the assignment of individuals to subpopulation membership regardless of the environment in which the analysis takes place (rainfed or full irrigation). 

## 3. Discussion

A comprehensive understanding of the genetic structure, and of how diversity is distributed, is needed to efficiently exploit and continuously advance in the wheat breeding programs to meet global future demands. Recent advances in genotyping and sequencing technologies have progressively expedited the introduction of genomic-based techniques in modern plant breeding. DArT, SSR, and SNP molecular marker systems have been validated for population genetic studies in wheat [25,26,27,28]. Particularly, Mora et al. [25] and, more recently, Ballesta et al. [28] reported that the population genetic structure of the Santa Rosa and Cauquenes sites is separated into two significantly differentiated clusters with relatively high differentiation values (Fst) according to the Bayesian clustering method (implemented in the software STRUCTURE) and a principal component analysis (PCA), which is in agreement with previous studies that have examined the genetic structure of different collections of bread wheat germplasm in the world [29,30]. Even though the cost of high-throughput genotyping has been decreasing over the years, it is still considered a limitation in the use of this technology in large-scale plant breeding. Genotyping thousands (or millions) of individuals is challenging and remains inaccessible for most species [31]. In fact, the cost efficiency of these tools has been improved considering the low coverage of the plant genomes and facilitated by genomic data imputation techniques [32]. The results of the present study revealed that the foliar spectral variability could be highly correlated with the genetic diversity of these wheat populations, which is demonstrated by the high classification accuracy (CA = 0.91) of the trained models from the spectral data (mainly, the neural network-based models). In general, the classification accuracy of the PLS-DA method was relatively high (CA > 0.7). However, it was less efficient than any of the neural networks tested in the present study (CNN and MLP). PLS-DA is a linear classifier, which has proven to be unsuitable for dealing with foliar spectral data that have several features to consider for classification procedures [33]. In fact, PLS-DA tends to make a decision criterion based on easily classifiable data, which leads to an inadequate classification for outliers [34]. The genetic structure of different plant populations has been inferred by non-supervised artificial neural network methods using SNP molecular markers [35,36]. Unlike previous studies, our approach used supervised classification neural network-based models, which consider foliar spectral reflectance information as “attributes” and the genetic structure (previously defined with SNPs) as the “class labels”. In general, CNN was significantly superior to MLP in terms of classification accuracy. According to Lowe et al. [37], despite the fact that CNN has a complex and time-consuming architecture for training the model, it is one of the methods with the highest precision and a high rate of object recognition. In this sense, CNN differs from MLP mainly by the existence of convulsion and pooling in CNN, which deals with the problem of dimensionality [38]. Additionally, MLP has a dense structure that significantly increases the number of parameters, which is not observable in CNN because it considers convulsion filters that gradually reduce the dimensionality of the data. 

Neural network models trained with foliar spectral data have been widely used to classify samples according to different criteria, such as phytosanitary, nutritional, and fruit development status, among others [39,40]. Consistent with the results of this study, Nachtigall et al. [39] evaluated the use of CNN and MLP to detect and classify diseases, damage, and nutritional deficiencies in apple trees from images of their leaves. The authors reported that the CNN models outperformed MLPs, achieving a classification accuracy of 97.3%. Moreover, both neural network models were compared with human expert perception, revealing that the MLP model was less efficient than expert perception in classifying diseases, damage, and nutritional deficiencies. It is noteworthy that, in the context of variety identification and genetic diversity studies, CNNs have proven to be more efficient than other traditional classification methods. Qiu et al. [41] classified rice seed varieties based on hyperspectral images using CNNs. According to the authors, the CNN model outperformed other methods, such as the K-nearest neighbors (KNN) and support vector machine (SVM) models, revealing the effectiveness of analyzing foliar spectral data based on CNN. In another study, Zhu et al. [42] modeled a CNN using near-infrared hyperspectral imaging to classify three varieties of soybeans (i.e., “Zhonghuang37”, “Zhonghuang41”, and “Zhonghuang55”), and each variety has a classification accuracy of over 90%. Kittlein et al. [43] performed a CNN model to provide a highly accurate prediction of the genetic diversity and differentiation of *Ctenomys australis* populations using molecular markers and high-resolution satellite imagery. CNN-based predictions accounted for about 98% of the variation observed in the genetic differentiation index and mean allele richness values, which may facilitate the identification of areas of interest for the conservation and management of populations. 

One of the most promising results of the present study is the fact that the neural networks achieved a classification accuracy higher than 80% for both environmental conditions (rainfed and full irrigation), which suggests that the ability of the genetic structure inference of neural network methods (based on foliar spectral data) was not significantly affected by environmental conditions. On the other hand, the results revealed that the classification accuracy was slightly lower in Cauquenes. Previous studies have discussed how water deficit interferes with the spectral signal of vegetation [44,45]. According to Damm et al. [44], plants subjected to water deficit exhibit higher reflectance values (especially within the shortwave infrared spectrum) than plants growing under higher water availability and may exhibit a higher variability in specific regions of the spectrum (in terms of the standard deviation). Therefore, rainfed conditions may be generating potential noise in spectral measurements, which in turn may be affecting classification models. Notably, CNNs exhibited the highest classification accuracy in both sites (Cauquenes and Santa Rosa), which can be explained by the fact that this method has a high ability to process data that have never before been observed and allows for robustness regarding data acquisition conditions, background heterogeneity, and intraclass variability [46]. It is important to note that, unlike neural network models, PLS-DA exhibited a higher classification accuracy in Cauquenes (rainfed) than in Santa Rosa (full irrigation), which indicates the potential of this methodology in assigning individuals to subpopulations under rainfed conditions.

**Figure 3:**
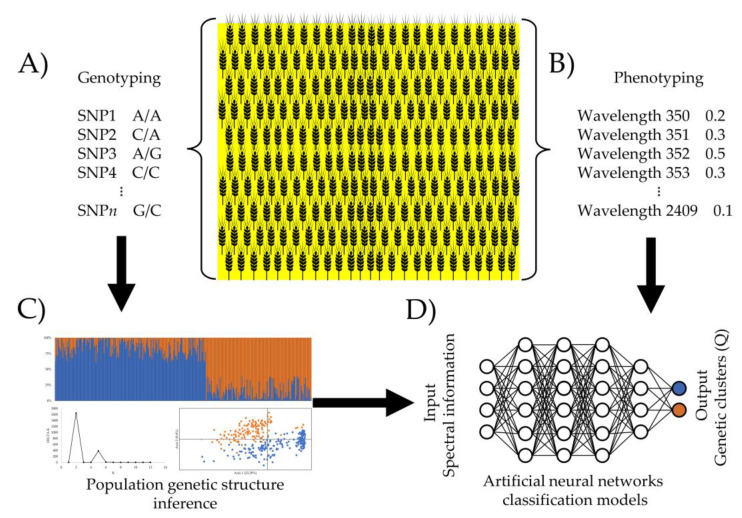
Overview of the spectral-based classification of genetically differentiated groups performed in this study. (**A**) Genotyping (using SNPs molecular markers) and (**B**) high-throughput phenotyping of the entire wheat panel (colored in yellow). (**C**) Molecular markers information is used to infer the population structure (cluster 1 (blue) and cluster 2 (orange)). (**D**) Training and validation of neural networks-based classification models (MLP and CNN).

Figure 3 provides an overview of the spectral-based classification of genetically differentiated groups carried out in this study. In brief, a spring wheat panel is genotyped using SNP markers, and then the genetic structure analysis is performed using the Bayesian clustering method (available in the STRUCTURE software) and Principal Components Analysis in order to confirm the genetic structure result. The genetic structure of the population is scored as an ordinal variable (in this wheat panel, clusters 1 and 2). Parallelly, high-throughput plant phenotyping of the entire population is performed using a specialized instrument. In this study, the foliar spectral data were considered input variables for the neural networks models [11], which are used to assign individuals to each genetically homogeneous group (clusters) previously inferred by the genetic structure analysis. Finally, the trained neural network models were used to assign individuals to a specific genetic cluster. It is necessary to consider that the analytical models of neural networks proposed in the present study are limited to classifying genotypes in genetic groups previously defined from the structure analysis; therefore, efforts should be made to obtain a genotyping for a set of individuals that are a good representation of the population diversity. The proposed methods are presented as a solution in the short term, at a low cost, and on a large scale of individuals. In addition, considering the relevance of examining the genetic structure of the population for breeding programs and the possibility of implementing high-throughput phenotyping in wheat panels, the pipeline based on neural network methods from foliar spectral data can be used to train neural network models to assign accessions to a given genetic subpopulation on a large scale and at a lower cost.

## 4. Materials and Methods

### 4.1. Plant Material and Experimental Design

A spectral-based classification study of genetically differentiated groups of wheat (*Triticum aestivum* L.) was carried out using a panel of 316 spring bread cultivars and advanced lines obtained from three breeding programs: the Agriculture Research Institute of Chile (INIA-Chile), INIA-Uruguay, and the International Wheat and Maize Improvement Center (CIMMYT). This panel was evaluated in two Mediterranean environment in Chile: Cauquenes (35°58′ S, 72°17′ W; 518 m.a.s.l.) and Santa Rosa (36°32′ S, 71°55′ W; 508 m.a.s.l.). Cauquenes is classified as a drought-prone area with a Mediterranean climate (with De Martonne arid index 20 ≤ IDM < 24) [28] and represents the water-limited conditions (rainfed). Santa Rosa exhibits a humid climate type (De Martonne index 28 ≤ MDI < 35) and represents a full irrigation condition [28]. The experimental design was an alpha-lattice design with 20 incomplete blocks per replicate and 20 genotypes per block.

### 4.2. High-Throughput Phenotyping

Spectral reflectance (350–2500 nm) was measured using a portable spectroradiometer (FieldSpec 3 Jr., ASD, Boulder, CO, USA), with a 2.3 mm diameter optical fiber. Measurements to 316 cultivars were made from 11:00 to 17:00 h. on clear days (radiation higher than 800 W m^−2^). The equipment was set up to take three scans per plot, with the bean of the optical fiber placed at 45° and 80 cm over the top of the canopy, and measurements were made by moving the sensor over the plot (covering the three central rows) according to Garriga et al. [47]. To limit variations in reflectance induced by changes in the angle of the sun, radiometric calibration was performed every 15 min, using a white barium sulfate panel as the reference (Spectralon, ASD, Boulder, CO, USA). The foliar spectral data were pre-processed in the R 4.0.5 software (Core Development Team, 2020), where the reflectance measures were normalized (centered and scaled), and their first derivative was computed using a Savitzky–Golay filter, with a window size of 37 data points.

### 4.3. High-Throughput Genotyping and Population Genetic Structure

DNA was isolated from the leaves of 316 cultivars according to the protocol of Porebski et al. [48]. Genotyping by sequencing (GBS) was carried out using the Illumina HiSeq 2000 sequencing platform and the bioinformatic approach for SNP identification described previously by Lado et al. [49]. SNP calls were made using the Tassel Pipeline (http://maizegenetics.net), with modifications for non-reference SNP calling according to Poland et al. [50] and Lado et al. [49]. SNPs with an MAF < 0.01 and a proportion of missing data per location >30% were eliminated according to Ballesta et al. [28], resulting in a total of 2,214 SNP markers from an initial dataset comprising 8,746 SNPs, which were used for the population genetic structure study.

The population genetic structure of the wheat was inferred using the STRUCTURE software [51], following Mora et al. [25]. For each genetically differentiated group (K, with K = 1–6), 10 runs were performed separately, each with 100,000 Monte Carlo Markov Chain (MCMC) replicates and a burn-in period of 10,000 iterations. The optimal K value was determined with Evanno’s method [24] according to Mora et al. [25]. The results of STRUCTURE are available in Ballesta et al. [28], which indicated that the 316 wheat cultivars were grouped into two genetically distinct subgroups (K = 2).

A description of the origin breeding program and respective genetic cluster for each cultivar is provided in Data Sheet S1, while the spectral signatures distinguishing each group are presented in Figure 2.

### 4.4. Classification Analysis

#### 4.4.1. Partial Least Squares Discriminant Analysis (PLS-DA)

PLS-DA is a regression approach where the reduction of the dimensions and the latent decomposition between a set of predictors X (feature matrix with I × J dimensions) and label responses Y (response with I × K dimensions) are key. PLS defines a new subspace of latent variables through an iterative process, considering a compromise between maximum variance in X and maximum correlation to Y [52]. This method can be described statistically by:

X = TP + E(1)

Y = UQ + F(2)

where the T and U matrices represent the score matrices X and Y, respectively, P and Q are the loading matrices of X and Y, respectively, and the E and F matrices correspond to the residual matrices of X and Y, respectively. The plsDA() function of the DiscriMiner package [53] in R 4.0.5 software [54] was used to run the PLS-DA algorithm.

#### 4.4.2. Neural Network Classifiers

In this study, two types of ANN were tested to classify spectral data: the first is plain Multi-Layer Perceptron (MLP), in which neurons in a layer are fully connected to all neurons in neighboring layers, and the second is a one-dimensional convolutional neural network (Conv1D), which is a special form of CNN. MLP is the simplest form of ANN, while Conv1D employs one-dimensional filters to capture the temporal pattern or shape of the input series.

The architecture of the CNN used in this study is based on a convolutional layer (conv1D) to realize the feature extraction of the data. The Conv1D-based classification models, in general, contain convolution layers, pooling layers, dense layers, and an output layer. In this sense, by applying different convolution filters within each convolution layer, the Conv1D has the ability to extract different one-dimensional features from different layers [55]. On the other hand, the pooling layers are generally used to provide samples with the input data by retaining only the maximum value of the feature map in a window with a specified pool size, while the dense layers represent a deeply connected neural network layer [56]. The Conv1D-based model used in this study was composed of two conv1D layers, a maximum one-dimensional sampling (MaxPooling1D) layer, a flatten layer (to transform the input data into a one-dimensional vector) with a dropout parameter (to reduce the overfitting of the training data), and, finally, a softmax layer which sets the predictions of the model (dense layer for classification). Because of the versatility of the specialized architectures, there is no standard procedure for searching for the optimal combination of hyper-parameters [56]. Therefore, in this study, the optimization of the Conv1D-based model was realized by combining different hyper-parameters. For this, the ReLU, Sigmoid, and Tanh activation functions were tested in conv1D, while the softmax activation function was used in the output layer. The categorical cross-entropy (categorical_crossentropy) was used as the loss function, and the Adam and rmsprop optimizers were tested. The training was realized with 500 epochs, testing a batch size of 10, 20, and 40 (Table 4).

MLP is a simple deep feedforward artificial neural network; there are at least three layers (input layer, hidden layer, and output layer), and the neurons of a layer are fully connected with all neurons of neighboring layers [57]. In this study, the architecture of MLP was composed of one or two dense hidden layers and an output layer (dense layer for classification). The ReLU, Sigmoid, and Tanh activation functions were tested in dense hidden layers, while softmax was in the output layer. categorical_crossentropy was used to track the loss function with the Adam and rmsprop optimizers (Table 4).

The two artificial neural network classification models were built and evaluated using Python v3.6.6, Tensorflow-gpu v1.13.1, and Keras v2.2.4.

#### 4.4.3. Performance Metric

The performance of the proposed CNN and MLP architectures was assessed in terms of classification accuracy, which measures the number of predictions correctly predicted divided by the total number of predictions made, multiplied by 100 (to express it as a percentage). This metric was used in the CNN and MLP networks.

Ten-fold cross-validation was applied for tuning each hyperparameter in the CNN and MLP networks and for the later comparison of the classification models (PLS-DA, CNN, and MLP). In each fold, the sample data were split into two datasets: training and validation sets, in which 80% of samples were randomly assigned to the training model, and the remaining 20% were used for validating the model. The Fisher LSD test was performed to compare the accuracy values among the evaluated models.

## 5. Conclusions

The present study evaluated the use of an artificial neural network-based spectral approach for the assignment of wheat genotypes to genetically differentiated groups (according to the genetic population structure), considering two contrasting water regimens. In general, the results revealed that the classification accuracy of neural networks was slightly lower in Cauquenes (rainfed condition). Notably, the clusters showed smaller differences in the wavelengths in Cauquenes than in Santa Rosa, which could have affected the average accuracy of the clustering in this environment. However, independent of environmental conditions (rainfed and full irrigation), the neural network models attain high classification accuracies. The methods proposed in this study can be applied to discriminate and assign accessions to a given genetic subpopulation in the short term and at a low cost, thus providing relevant information for breeding purposes. Additionally, the results demonstrated that this methodology has a high precision in different environmental conditions, which is highly desirable in the context of selection methodologies (e.g., marker-assisted selection) and studies related to the conservation of germplasm with a high diversity.

## Figures and Tables

**Table 1:** Classification accuracy of the population structure in wheat for 36 MLP and CNN models, considering different hyperparameters: activation function (ReLU, Tanh, and Sigmoid), N° layer (one or two dense layers in MLP—one or two extra dense layers in CNN), optimizer (adam or rmsprop), and batch size (10, 20, and 40).

Activation Function	N° Layer	Optimizer	Batch Size	MLP		CNN	
				SR	CAU	SR	CAU
ReLU	1	adam	10	0.91 *	0.78	0.91 *	0.89 *
			20	0.87	0.88 *	0.86	0.84
			40	0.80	0.78	0.86	0.87
		rmsprop	10	0.81	0.82	0.83	0.82
			20	0.83	0.86	0.80	0.84
			40	0.83	0.72	0.82	0.82
	2	adam	10	0.88	0.83	0.76	0.81
			20	0.83	0.85	0.87	0.82
			40	0.90	0.81	0.83	0.86
		rmsprop	10	0.88	0.76	0.82	0.80
			20	0.80	0.70	0.85	0.78
			40	0.63	0.81	0.82	0.83
sigmoid	1	adam	10	0.71	0.60	0.73	0.75
			20	0.82	0.62	0.76	0.68
			40	0.85	0.77	0.69	0.68
		rmsprop	10	0.71	0.75	0.77	0.85
			20	0.73	0.83	0.81	0.84
			40	0.81	0.61	0.75	0.80
	2	adam	10	0.79	0.83	0.65	0.68
			20	0.82	0.77	0.70	0.68
			40	0.83	0.79	0.67	0.62
		rmsprop	10	0.76	0.68	0.72	0.75
			20	0.68	0.74	0.65	0.76
			40	0.65	0.66	0.64	0.68
tanh	1	adam	10	0.71	0.61	0.79	0.76
			20	0.76	0.79	0.82	0.85
			40	0.81	0.83	0.83	0.85
		rmsprop	10	0.69	0.64	0.61	0.86
			20	0.83	0.74	0.59	0.38
			40	0.65	0.84	0.49	0.66
	2	adam	10	0.70	0.62	0.81	0.87
			20	0.69	0.69	0.83	0.83
			40	0.85	0.81	0.81	0.81
		rmsprop	10	0.64	0.67	0.67	0.71
			20	0.62	0.61	0.63	0.76
			40	0.79	0.60	0.59	0.55

MLP: Multilayer Perceptron; CNN: Convolutional Neural Network; SR: Santa Rosa; CAU: Cauquenes; * Highest classification accuracy among all hypermeters.

**Table 2:** Average classification accuracy of each hyperparameter in the classification of the population structure of wheat through MLP and CNN models.

Hyperparameter		CNN		MLP	
		SR	CAU	SR	CAU
Activation function	ReLU	0.836 a	0.832 a	0.831 a	0.800 a
	sigmoid	0.712 b	0.731 b	0.763 b	0.721 b
	tanh	0.706 b	0.741 b	0.728 b	0.704 b
N° layer	1	0.762 a	0.780 a	0.785 a	0.748 a
	2	0.740 a	0.756 a	0.763 a	0.735 a
Optimizer	adam	0.788 a	0.786 a	0.807 a	0.759 a
	rmsprop	0.714 b	0.749 a	0.741 b	0.724 a
Batch size	10	0.756 a	0.796 a	0.766 a	0.716 a
	20	0.764 a	0.755 a	0.773 a	0.757 a
	40	0.733 a	0.753 a	0.783 a	0.753 a

MLP: Multilayer Perceptron; CNN: Convolutional Neural Network; SR: Santa Rosa; CAU: Cauquenes. Different letters show the statistical significance within each hyperparameter at a *p*-value < 0.01 using the Fisher LSD test.

**Table 3:** Summary of the results of the classification accuracy on the test dataset for 10 repetitions in the wheat population in two environments.

Environment	Model	Accuracy ± SD	Accuracy Cluster 1 ± SD	Accuracy Cluster 2 ± SD
Santa Rosa (full irrigation)	MLP	0.92 ± 0.01 bA	0.94 ± 0.02 aA	0.89 ± 0.03 bA
	CNN	0.93 ± 0.01 aA	0.93 ± 0.02 aA	0.95 ± 0.03 aA
	PLS-DA	0.74 ± 0.02 cB	0.80 ± 0.08 bB	0.70 ± 0.08 cA
Cauquenes (rainfed)	MLP	0.88 ± 0.01 bB	0.92 ± 0.02 aA	0.82 ± 0.03 bB
	CNN	0.92 ± 0.01 aA	0.95 ± 0.02 aA	0.89 ± 0.03 aB
	PLS-DA	0.78 ± 0.02 cA	0.85 ± 0.08 bA	0.73 ± 0.08 cA
Individuals in cluster		316	186	130

SD: Standard Deviation; MLP: Multilayer Perceptron; CNN: Convolutional Neural Network; PLS-DA: Partial Least-Squares Discriminant Analysis. Statistical significance between different models (PLS-DA, MLP and CNN) in each environment is noted by lowercase letters, while the statistical significance for each model between different environments is shown by uppercase letters. Different letters show the statistical significance at a *p*-value < 0.01 using the Fisher LSD test.

**Table 4:** Values used for each hyperparameter in this study.

Parameter	Values Used
Activation functions	ReLU, Tanh, and Sigmoid
Number of layers	MLP: one or two dense layers; CNN: one or two conv1D layers
Optimizer algorithm	Adam and rmsprop
Batch size	10, 20, and 40

## Data Availability

The data presented in this study are available upon request from the corresponding author.

## Supplementary Materials

The following supporting information can be downloaded at: https://www.mdpi.com/article/10.3390/plants12030440/s1, Data Sheet S1: Description of the origin breeding program and the respective genetic cluster for each spring wheat cultivar (from genetic structure analyses).
